# ARTS AND HUMANITIES BRIDGING ACROSS CONTINUUM OF CARE: CASE STUDY, GEORGETOWN LOMBARDI COMPREHENSIVE CENTER

**DOI:** 10.1093/geroni/igz038.114

**Published:** 2019-11-08

**Authors:** Julia Langley

**Affiliations:** Georgetown Lombardi Comprehensive Cancer Center, Washington, District of Columbia, United States

## Abstract

How does a hospital-based arts and humanities program build towards a better healthcare system for older patients, caregivers and medical professionals? The Georgetown Lombardi Arts and Humanities Program (AHP) supports the continuum of care by encouraging a creative and constructive response to illness through the use of music, dance, expressive writing and visual arts. Artists-in-residence at MedStar Georgetown University Hospital work throughout the hospital, with in-patients and out-patients, in waiting rooms, intensive care units, and clinics. At the same time, the AHP recognizes the need to introduce the next generation of physicians, nurses, and medical professionals to the benefits of interacting with the arts to improve skills of observation, communication, empathy, and perspective-taking to serve all populations, especially older patients living with chronic illness. The AHP’s educational programs at the National Gallery of Art, and the IONA Senior Day Center will be highlighted along with their supporting evidence base

